# How Do Urban Indian Private Practitioners Diagnose and Treat Tuberculosis? A Cross-Sectional Study in Chennai

**DOI:** 10.1371/journal.pone.0149862

**Published:** 2016-02-22

**Authors:** Liza Bronner Murrison, Ramya Ananthakrishnan, Sumanya Sukumar, Sheela Augustine, Nalini Krishnan, Madhukar Pai, David W. Dowdy

**Affiliations:** 1 Department of Epidemiology, Johns Hopkins Bloomberg School of Public Health, Baltimore, Maryland, United States of America; 2 Center for Tuberculosis Research, Johns Hopkins University, Baltimore, Maryland, United States of America; 3 REACH, Chennai, India; 4 McGill International TB Centre & Department of Epidemiology, Biostatistics, and Occupational Health, McGill University, Montreal, Quebec, Canada; Indian Institute of Science, INDIA

## Abstract

**Setting:**

Private practitioners are frequently the first point of healthcare contact for patients with tuberculosis (TB) in India. Inappropriate TB management practices among private practitioners may contribute to delayed TB diagnosis and generate drug resistance. However, these practices are not well understood. We evaluated diagnostic and treatment practices for active TB and benchmarked practices against International Standards for TB Care (ISTC) among private medical practitioners in Chennai.

**Design:**

A cross-sectional survey of 228 practitioners practicing in the private sector from January 2014 to February 2015 in Chennai city who saw at least one TB patient in the previous year. Practitioners were randomly selected from both the general community and a list of practitioners who referred patients to a public-private mix program for TB treatment in Chennai. Practitioners were interviewed using standardized questionnaires.

**Results:**

Among 228 private practitioners, a median of 12 (IQR 4–28) patients with TB were seen per year. Of 10 ISTC standards evaluated, the median of standards adhered to was 4.0 (IQR 3.0–6.0). Chest physicians reported greater median ISTC adherence than other MD and MS practitioners (score 7.0 vs. 4.0, P<0.001), or MBBS practitioners (score 7.0 vs. 4.0, P<0.001). Only 52% of all practitioners sent >5% of patients with cough for TB testing, 83% used smear microscopy for diagnosis, 33% monitored treatment response, and 22% notified TB cases to authorities. Of 228 practitioners, 68 reported referring all patients with new pulmonary TB for treatment, while 160 listed 27 different regimens; 78% (125/160) prescribed a regimen classified as consistent with ISTC. Appropriate treatment practices differed significantly between chest physicians and other MD and MS practitioners (54% vs. 87%, P<0.001).

**Conclusion:**

TB management practices in India’s urban private sector are heterogeneous and often suboptimal. Private providers must be better engaged to improve diagnostic capacity and decrease TB transmission in the community.

## Introduction

India accounts for approximately one-quarter of the world’s 9 million incident tuberculosis (TB) cases every year [1]. The TB epidemic in India is complicated by the fragmented healthcare delivery system that includes practitioners in the public and private sectors [1–3]. Although the Government of India’s Revised National TB Control Program (RNTCP) provides free TB healthcare services, up to 85% of patients experiencing TB-related symptoms in urban Indian settings first seek healthcare from private practitioners [3,4], and about 50% ultimately get TB treatment outside the RNTCP [5,6].

Based on the International Standards for TB Care (ISTC) [7], India has recently published Standards for TB Care in India to ensure quality practices in both private and public sectors [8]. However, usage of inappropriate TB diagnostic and treatment practices, and lack of adherence to the ISTC continues to be documented among private practitioners in India [5,6,9–11], potentially contributing to delays in TB diagnosis, development of drug resistance, and ongoing TB transmission [6,10,12].

Currently, there are several ongoing efforts to engage and improve the quality of TB care in the private sector. These include NGO-led models [13] and Private Provider Interface Agency projects in select cities [14] to engage private practitioners in TB care and the Initiative for Promoting Affordable Quality TB Tests (IPAQT) to increase availability of appropriate diagnostic tests in private laboratories [12,15]. In this context, it is helpful to assess urban private practitioners’ practices for diagnosing and treating patients with active pulmonary TB.

## Methods

### Setting

Chennai is the sixth largest city in India with a population of 4.6 million [16]. There are an estimated 550 hospitals registered in Chennai and approximately 10,000 doctors working in the city, though the public-private affiliations are unknown [17]. We collaborated with the Resource Group for Education and Advocacy for Community Health (REACH) to conduct a cross-sectional survey of private medical practitioners (PPs) who saw at least one patient with TB in the past year. Established in 1999, REACH is a non-governmental public-private mix (PPM) organization collaborating with the Corporation of Chennai to involve PPs in the RNTCP [13]. REACH works with a network of PPs that refer patients with TB to their four PPM Centers, located in private hospitals in Chennai, to receive free TB treatment under supervision of REACH staff or community supporters.

### Sample selection and data collection

We recruited qualified, allopathic medical practitioners of all specialties working in the urban private sector. From January 2014 through February 2015, we enrolled consenting PPs from the ten 2011 census tract zones in Chennai and conducted interviews using a structured questionnaire. To identify PPs for recruitment, we used a listing of all city streets in Chennai to randomly select up to 50 street corners per zone. REACH staff started at each selected corner and used a structured movement algorithm until an eligible PP was located to recruit a maximum of five PPs per health facility and minimum of five PPs per zone. Additionally, we randomly selected PPs from REACH’s database of PPM-referring PPs. All qualified PPs with formal medical training who diagnosed at least one patient with pulmonary TB (PTB) in the year prior to recruitment were eligible. After a PP agreed to be interviewed for the study, up to three attempts were made to complete the interview. Trained study staff performed the interviews privately in the PPs’ offices. Structured interviews lasted approximately 15–20 minutes and collected data on PPs’ self-reported socio-demographic information (excluding age, which was deemed potentially identifiable during ethical review), patient volume, TB disease knowledge, diagnostic and treatment practices, intervals between patient presentation, diagnosis, and treatment, and patient referral practices. PPs were asked about the drug regimen that they prescribed for their new adult patients of 60 kilograms with PTB using an open-ended format. PPs were subsequently asked to give the average total duration of treatment for PTB from a set of standardized responses. PP-reported education was used to assess level of training by comparing those with higher levels of training (i.e., medical doctor (MD) or master of surgery degree (MS)), to those with undergraduate bachelor of medicine and bachelor of surgery degrees (MBBS). We also compared chest specialists against those without such specialty training.

### International standards for TB care (ISTC) 3^rd^ Edition

The ISTC include 21 standards of diagnosis, treatment, and other practices that describe a widely accepted level of TB care [7,10]. We evaluated PP-reported practices against 10 ISTC standards (those for which comparative data were collected). An ISTC score was calculated by summing the total number of standards for which PP-reported practices agreed with ISTC recommendations. For example, a score of seven means the PP reported practices that were in accordance with seven of the 10 standards evaluated.

### Statistical analysis

Our primary comparisons were of PPs seeing >12 TB patients per year (versus ≤12), and of MD and MS specialty physicians versus MBBS practitioners. We estimated estimate univariate and multivariable associations of self-reported volume of TB patients with provider specialty, usage of diagnostic tests, and adherence to ISTC using Poisson regression with robust standard errors, as the outcome was not rare and log-binomial models failed to converge [18]. These models also included a term for each provider’s health facility in order to account for clustering by facility. We explored differences in mean patient volume using generalized linear models assuming gamma-distributed residuals, identity link function, and robust standard errors. We also estimated multivariable associations with self-reported rapid TB diagnosis (≤7 days after patient presentation) using Poisson regression models to give relative risks. Differences in median ISTC score were explored using the nonparametric Wilcoxon test and differences in mean score using linear regression models with robust standard errors. We examined collinearity for all models using variance inflation factors, which were below 3.0 for all covariates included in the model.

### Ethical considerations

This study protocol was approved by the Johns Hopkins Bloomberg School of Public Health Institutional Review Board, McGill University Health Center Biomedical Research Ethics Review Board, and REACH Independent Ethics Committee. Written informed consent was obtained from all practitioners prior to being interviewed for the study.

## Results

### Practitioner socio-demographic profiles

Of the 249 eligible private practitioners approached to participate, 228 were interviewed (92% participation rate). Among these, 161 (71%) were randomly selected within Chennai city and 67 (29%) from REACH’s PPM database. The majority of PPs were men (70%) and median practice experience was 20 years (IQR 15–30) (Table 1). Over half (60%) of PPs had medical degrees and 36% had MBBS undergraduate degrees; 17% were chest specialist physicians. Overall, 56% of PPs worked in private standalone clinics, 34% in private hospitals, and 10% in both government and private healthcare settings.

**Table 1:** Socio-demographic profile and management practices for all forms of TB among private practitioners in Chennai, India (n = 228).

		Patients Diagnosed with TB in Past Year							
Characteristic	Total (n = 228) n(%)	≤12 Patients (n = 110) n(%)	>12 Patients (n = 118) n(%)	Unadjusted PR (95% CI)†	*P*	Adjusted PR (95% CI)^1^	*P*^1^	Adjusted PR (95% CI)^2^	*P*^2^
Male	160 (70)	67 (61)	93 (79)	**1.6 (1.1–2.3)**	**0.01**	1.2 (0.9–1.7)	0.23	1.3 (0.9–1.8)	0.21
Education									
MBBS	81 (35)	53 (48)	28 (24)	REF		REF		REF	
MD (General internal medicine)	11 (5)	5 (4)	6 (5)	1.1 (0.6–1.8)	0.84	1.3 (0.9–2.0)	0.21	1.5 (0.8–2.6)	0.19
MD (Chest/Pulmonary specialty)	39 (17)	6 (6)	33 (28)	**1.9 (1.5–2.4)**	**<0.001**	**1.9 (1.4–2.6)**	**<0.001**	**2.3 (1.5–3.5)**	**<0.001**
MD (Other specialty)	86 (38)	42 (38)	45 (38)	1.0 (0.8–1.3)	0.99	1.4 (0.9–2.1)	0.10	**1.6 (1.0–2.5)**	**0.04**
MS	11 (5)	4 (4)	6 (5)	1.2 (0.7–2.1)	0.60	1.6 (0.8–3.2)	0.19	1.7 (0.8–3.7)	0.17
Years practicing, *median [IQR]*	20 [15–30]	20 [12–30]	25 [15–30]	**1.0 (1.0–1.0)**	**0.03**	**1.0 (1.0–1.0)**	**<0.001**	**1.0 (1.0–1.0)**	**0.01**
Practitioner specialty									
General medicine	155 (68)	85 (77)	70 (59)	REF		REF		REF	
Chest/Pulmonary specialist	37 (16)	4 (4)	33 (28)	**1.9 (1.5–2.4)**	**<0.001**	Omitted		**1.7 (1.3–2.1)**	**<0.001**
Other*	36 (16)	21 (19)	15 (13)	0.8 (0.5–1.2)	0.21	0.8 (0.5–1.2)	0.30	0.8 (0.5–1.2)	0.22
Type of facility									
Government with private practice in evening	22 (10)	13 (12)	9 (8)	REF		REF			
Private standalone clinic or polyclinic	129 (56)	76 (69)	53 (45)	**0.6 (0.5–0.8)**	**<0.001**	--			
Private hospital or nursing home	77 (34)	21 (19)	56 (47)	**1.8 (1.4–2.3)**	**<0.001**	--			
Action for pulmonary TB diagnosis									
Refer to RNTCP or PPM DOTS center	97 (42)	55 (50)	42 (36)	REF		REF			
Treatment in private sector	131 (58)	55 (50)	76 (64)	**1.3 (1.0–1.8)**	**0.05**	1.1 (0.8–1.4)	0.66		
Knowledge of TB notification requirement**	214 (94)	99 (90)	115 (98)	2.5 (0.8–8.0)	0.12	1.7 (0.7–4.5)	0.26		
Notification training	118 (52)	39 (36)	79 (67)	**1.9 (1.5–2.4)**	**<0.001**	**1.5 (1.2–2.0)**	**<0.01**		
Notified RNCTP of any TB patients	49 (22)	20 (18)	29 (25)	1.2 (0.9–1.6)	0.25	1.0 (0.7–1.3)	0.84		
Knowledge of serological antibody test ban	126 (55)	48 (44)	78 (66)	**1.6 (1.1–2.3)**	**0.02**	1.2 (0.9–1.6)	0.21		
Awareness of PPM schemes via RNTCP	81 (35)	37 (34)	44 (37)	1.1 (0.8–1.5)	0.66	0.9 (0.7–1.2)	0.40		
Source of information on TB**									
No sources/not PP's specialty	39 (17)	28 (16)	11 (9)	**0.5 (0.3–0.9)**	**0.02**	**0.4 (0.2–0.8)**	**<0.01**		
Journals, books, newspaper, newsletters	82 (36)	36 (33)	46 (39)	1.1 (0.9–1.4)	0.28	**0.8 (0.6–1.0)**	**0.03**		
Internet	71 (31)	33 (30)	38 (32)	1.1 (0.8–1.3)	0.66	0.9 (0.7–1.2)	0.45		
CME or workshop	99 (43)	45 (41)	54 (46)	1.1 (0.8–1.5)	0.55	**0.7 (0.5–1.0)**	**0.03**		
Medical representative or colleague	25 (11)	15 (14)	10 (9)	0.8 (0.4–1.3)	0.33	0.7 (0.4–1.2)	0.24		

MD, Medical doctor degree; MS, Master of surgery degree; MBBS, Bachelor of medicine and bachelor of surgery undergraduate degrees; RNTCP, Revised National TB Control Program; PPM DOTS, Public-Private Mix directly observed therapy short course.

*Other MD and MS practitioner specialties included Obstetrics and gynecology (n = 15), Pediatrician (n = 5), Surgeon (General/Orthopedic/Ophthalmologic) (n = 4), Diabetes Specialist (n = 6), Ear nose and throat (n = 2), Oncologist (n = 1), and Radiologist (n = 1).

**Categories are not mutually exclusive.

†Unadjusted prevalence ratio comparing practitioners with annual TB patient volumes of at least 12 patients versus greater than 12 patients with TB per year.

^1^Prevalence ratio adjusted for all variables with data in this column. Adjusted PR for pulmonary specialist omitted due to overlap with education. Variance inflation factors were below 3.0 for all covariates included in the model.

^2^Prevalence ratio adjusted for sex, education, years practicing, and specialty. Variance inflation factors were below 3.0 for all covariates included in the model.

**Fig 1:**
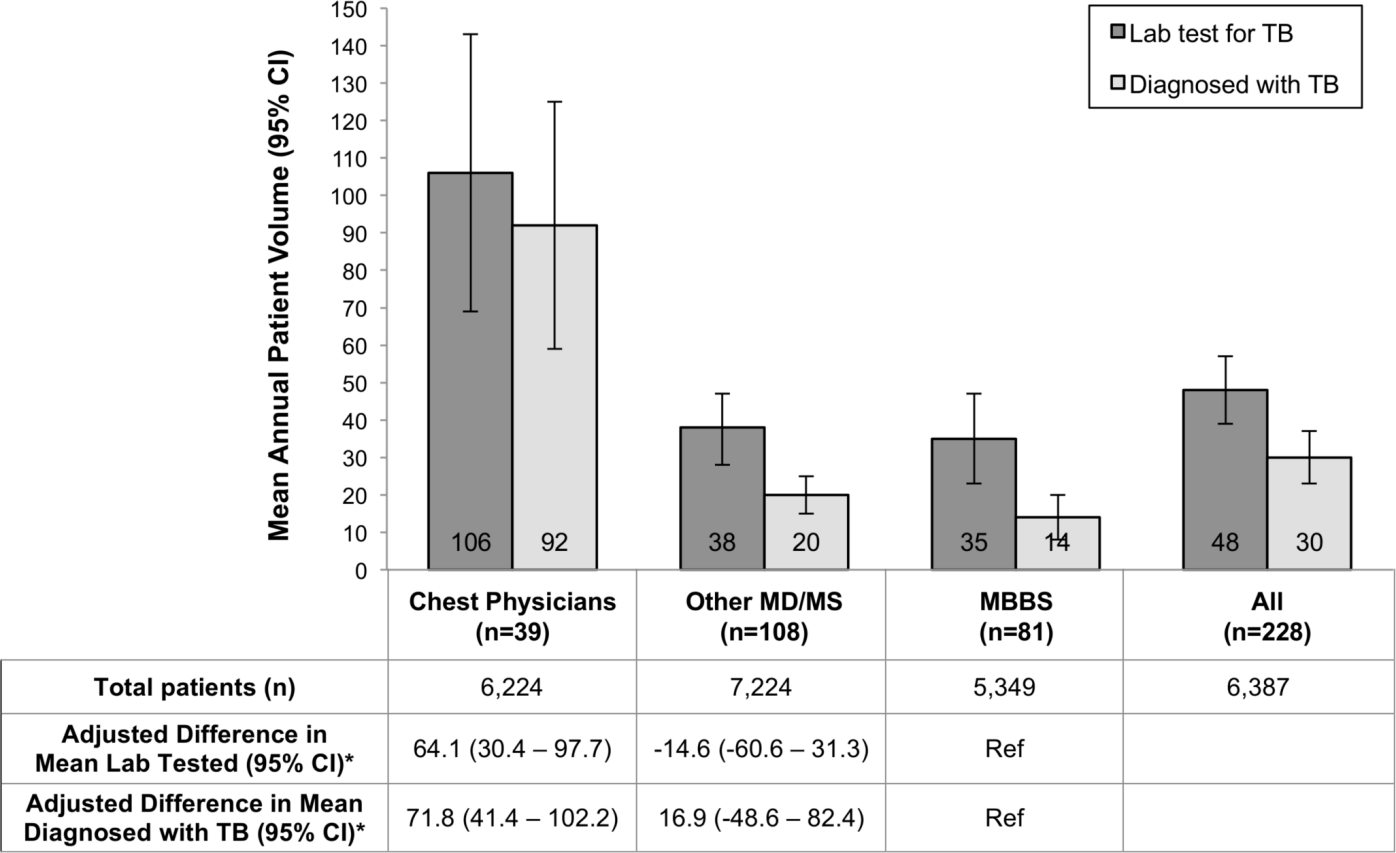
Mean annual volume of patients with tuberculosis (TB) in the past year according to practitioner training among private practitioners in Chennai. Dark bars show the mean (with 95% confidence intervals shown as error bars) number of patients for whom a laboratory test was sent specifically to diagnose TB in the past year, as reported by private practitioners with each level of training. Light bars show the mean number of people diagnosed with TB in that year. Data table shows the mean number of total patients seen in the past year.

The mean annual patient volume in our sample was 6,387 (median 6,000 [IQR 4,000–8,000]) and 30 patients with TB (median 12 [IQR 4–28]). Chest physicians saw a mean of 92 (95%CI: 59–125) TB patients per year, versus 20 (95%CI: 15–25) among other MD/MS physicians, and 14 (95%CI: 8–20) among MBBS practitioners (Fig 1). Chest physicians reported diagnosing significantly more patients with TB per year than MBBS practitioners (mean patients: 92 vs. 14; adjusted mean difference: 71.8; 95%CI: 41.1–102.2).

In univariate analysis, providers who saw more than 12 patients with TB per year were more likely to be specialists, have more years of practice experience, work in a private hospital, initiate TB treatment without referral to the public sector, know about Indian TB policies, and obtain knowledge about TB from such sources as medical journals (Table 1). In adjusted analyses, those providers who saw at least 12 TB patients were significantly more likely to be chest specialists (aPR: 1.9, 95%CI: 1.4–2.6) and to report knowledge of Indian TB notification policies (aPR: 1.5, 95%CI: 1.2–2.0), while being less likely to report no sources of knowledge on TB (aPR 0.4, 95%CI: 0.2–0.8).

### Diagnostic testing practices

Sputum smear microscopy was commonly used by all providers for diagnosis of active TB (83%; 95%CI: 77–87), as was chest X-ray (97%; 95%CI: 95–99). Practitioners frequently relied on non-microbiological tests not generally recommended for active TB diagnosis as part of their diagnostic process including erythrocyte sedimentation rate (ESR) (54%; 95%CI: 45–60) and tuberculin skin testing (TST; 45%; 95%CI: 39–52) (Table 2). Over one-third (39%) reported using only smear and chest X-ray for diagnosis of active pulmonary TB with or without ESR; 17% relied on only smear, chest X-ray, and ESR in addition to TST. Molecular testing (e.g., Xpert MTB/RIF) and TB culture were much less commonly employed (15%; 95%CI: 11–20, and 15%; 95%CI: 10–19, respectively). Usage of serological antibody tests and IGRAs for diagnosis of active PTB was reportedly low among all practitioners (2%; 95%CI: 0–4, and 6%; 95%CI: 3–9, respectively).

**Table 2:** Diagnostic testing practices according to practitioner specialty type among private practitioners in Chennai, India (n = 228).

		Used in TB diagnosis n(%)						Ordered at first patient visit n(%)							
			Practitioner Level of Training							Practitioner Level of Training					
Diagnostic Test	Mean (SD) tests per month	All (n = 228)	Chest Physicians (n = 39)	Other MD/MS (n = 108)^†^	MBBS (n = 81)	*P*‡*		All (n = 228)		Chest Physicians (n = 39)		Other MD/MS (n = 108)^†^		MBBS (n = 81)	*P*‡*
Chest X–Ray	3.2 (4)	222 (97)	36 (92)	106 (98)	80 (99)	0.43		135 (59)		31 (80)		64 (59)		40 (49)	**0.03**
Sputum Smear	3.5 (4)	188 (83)	36 (92)	91 (84)	61 (75)	0.04*		115 (50)		31 (80)		54 (50)		30 (37)	<0.01*
Biopsy or FNAC	1.2 (3)	150 (66)	34 (87)	80 (74)	36 (44)	<0.001*		108 (47)		31 (80)		53 (49)		24 (30)	<0.001*
ESR	3.2 (4)	122 (54)	12 (31)	70 (65)	40 (49)	0.35		68 (30)		9 (23)		41 (38)		18 (22)	0.06
Mantoux Skin Test	2.4 (4)	103 (45)	12 (31)	50 (46)	41 (51)	0.22		61 (27)		12 (31)		29 (27)		20 (25)	0.60
Differential or Total Lymphocyte Count	4.1 (4)	71 (31)	8 (21)	45 (42)	18 (22)	0.03*		45 (20)		7 (18)		28 (26)		10 (12)	**0.04**
MRI or CT Scan	1.1 (4)	53 (23)	17 (44)	26 (24)	10 (12)	<0.01*		39 (17)		14 (36)		16 (15)		9 (11)	0.07
Molecular Test (Xpert or PCR)	1.0 (4)	35 (15)	23 (59)	11 (10)	1 (1)	<0.001*		28 (12)		22 (56)		5 (5)		1 (1)	<0.001*
Sputum Culture	1.4 (2)	33 (15)	9 (23)	9 (8)	15 (19)	0.20		25 (11)		7 (18)		7 (7)		11 (14)	0.35
Ultrasound	0.4 (1)	32 (14)	5 (13)	17 (16)	10 (12)	0.59		24 (11)		5 (13)		13 (12)		6 (7)	0.37
Drug Susceptibility Testing	0.8 (3)	19 (8)	16 (41)	2 (2)	1 (1)	<0.01*		17 (8)		14 (36)		2 (2)		1 (1)	<0.01*
Interferon Gamma Release Assays	2.8 (4)	14 (6)	3 (8)	7 (7)	4 (5)	0.78		5 (2)		1 (3)		3 (3)		1 (1)	0.66
Serologic Antibody Tests	1.0 (1)	5 (2)	1 (3)	1 (1)	3 (4)	0.35	2 (1)			0 (0)		1 (1)		1 (1)	0.99

MD, Medical doctor degree; MS, Master of surgery degree; MBBS, Bachelor of medicine and bachelor of surgery undergraduate degrees; FNAC, fine needle aspiration cytology; ESR, erythrocyte sedimentation rate; MRI, magnetic resonance imaging; CT, computerized tomography; Xpert, Xpert MTB/RIF; PCR, polymerase chain reaction.

†Other MD/MS practitioner specialties included Other/unknown (n = 61), Obstetrics and gynecology (n = 15), General internal medicine (n = 11), Pediatrician (n = 5), Surgeon (General/Orthopedic/Ophthalmologic) (n = 4), Diabetes Specialist (n = 6), Ear nose and throat (n = 2), Oncologist (n = 1), and Radiologist (n = 1).

‡Pearson's chi-squared (or Fisher's exact) test for categorical variables comparing chest physicians plus other MD/MS practitioners versus MBBS practitioners in the private sector.

*Remains significant risk factor (P≤0.05) for use of test in TB diagnosis comparing practitioner level of training after adjustment for sex, years practicing, facility type, and total patient volume.

Chest physicians and other MD/MS practitioners were more likely to report using smear microscopy (86% vs. 75%, P = 0.04) and Xpert MTB/RIF (23% vs. 1%, P<0.001) for TB diagnosis than MBBS practitioners. In adjusted analyses, chest physicians and other MD/MS providers were significantly more likely to use smear, Xpert, and DST in the TB diagnostic process or at the first patient visit than MBBS providers (Table 2). In a comparison of shorter versus longer PP-reported delays (≤7 days vs. >7 days) from patient presentation to TB diagnosis, the use of smear microscopy as the initial diagnostic test was the only variable associated with more rapid PP-reported diagnosis, with a relative risk of 3.2 (95%CI: 2.3–4.3) for diagnosis within one week.

Among those practitioners that reported using smear, Xpert, and culture for TB diagnosis, 57% (107/228), 83% (29/35), and 55% (18/33) of practitioners, respectively, reported sending patients to private labs for diagnostic testing. The majority of practitioners reported sending patients to private labs for other diagnostic tests including: 65% (144/222) for chest X-ray, 70% (85/122) for ESR, 73% (75/103) for TST, and 93% (13/14) for IGRAs. The main reason given by all PPs for using smear and chest X-ray were test accuracy (34% and 51%), while the main problem with existing diagnostic tests reported was the necessity of sputum samples (30%).

### ISTC and TB treatment practices

Adherence to the ISTC was generally inadequate (Table 3) with an overall median ISTC adherence score of 4.0 (IQR 3.0–6.0) out of ten standards evaluated. Among PPs who saw patients with cough lasting >2 weeks in the week prior to being interviewed, only 52% sent more than 5% of these patients for TB laboratory testing. Only 25% of all PPs used culture or molecular testing for patients with clinical suspicion of TB, 33% monitored treatment response, and 22% notified TB cases to public authorities. There were no significant differences in use of smear microscopy by patient volume, health facility type, or health sector. Chest physicians and other MD/MS practitioners with higher levels of training reported greater adherence to all standards except for direct observation of treatment (Table 3). In analyses adjusted for sex, practice years, facility type, and patient volume, the prevalence of ISTC adherence for use of smear for PTB, examination of appropriate specimens for presumptive EP TB, and drug susceptibility testing was greater among chest physicians and other MD/MS practitioners compared to MBBS providers.

**Table 3:** Evaluation of private practitioners' practices and concordance with the International Standards for TB Care, Chennai, India.

		Concordance with ISTC n(%)				
			Practitioner Level of Training			
International Standards for TB Care	Mechanism for evaluation	All (n = 228)	Chest Physicians (n = 39)	Other MD/MS (n = 108)	MBBS (n = 81)	P**°
**Diagnostic Practices**	** **					
**ISTC 1:** Evaluation of cough lasting ≥2 weeks to suspect TB	*Sends >5% patients with cough >2 weeks for lab testing*	93 (52)	23 (73)	38 (44)	32 (53)	0.77
**ISTC 2**: Evaluation of sputum specimens for those with CXR findings suggestive of TB	*Uses smear and chest x-ray for PTB diagnosis*	185 (81)	35 (90)	89 (82)	61 (75)	0.09
**ISTC 3:** Assessment of ≥2 sputum specimens for microbiological examination	*Uses smear for PTB*	188 (83)	36 (92)	91 (84)	61 (75)	**0.04**°
**ISTC 4**: Examination of appropriate specimens (and diagnostic tests) for presumptive EP TB	*Uses biopsy/FNAC to obtain specimens for EP-TB diagnostic testing*	150 (66)	34 (87)	80 (74)	36 (44)	**<0.001**°
**ISTC 5**: Diagnosis of smear-negative TB based on bacterial culture or molecular testing	*Uses culture or Xpert MTB/RIF for TB diagnosis*	57 (25)	25 (64)	16 (15)	16 (20)	0.17
**Treatment and Management Practices**	*** ***					
**ISTC 8**: Treatment with 2HRZE and 4HR fixed doses for new TB patients*†Ŧ	*Regimen of H*, *R*, *and either Z or E for 6–8 months for hypothetical patient with new PTB*	125 (78)	20 (54)	67 (87)	38 (83)	0.41
**ISTC 9**: Patient-centered approach to treatment; includes DOTS for treatment administration	*Supported treatment via RNTCP*, *PPM*, *or PPM Community DOTS reported for PTB*	92 (40)	3 (8)	39 (36)	50 (62)	**<0.001**°
**ISTC 10**: Assessment of patient response to therapy for pulmonary TB with sputum microscopy	*Performs treatment monitoring using smear*, *culture*, *or Xpert MTB/RIF*	76 (33)	27 (69)	23 (21)	26 (32)	0.77
**ISTC 11**: Drug-resistance testing using molecular tests and/or bacterial culture based on patient history and risk factors	*Assesses MDR based on appropriate risk factors*	43 (19)	29 (74)	8 (7)	6 (7)	**<0.001**°
**Addressing HIV Infection and other Co-morbid Conditions**						
**ISTC 14**: HIV testing and counseling recommended to all patients with suspected TB	*Performs HIV testing done Always or Often for TB patients*	87 (38)	25 (64)	34 (32)	28 (35)	0.41
**Standards for Public Health**						
**ISTC 21:** All providers must report TB cases and treatment outcomes to public health authorities.	*Reports notifying TB cases to RNTCP in the past year*	49 (22)	16 (41)	16 (15)	17 (21)	0.89
Mean ISTC score, mean(SD)**Ŧ	*Sum out of 10 standards evaluated*	4.5 (1.9)	6.5 (2.0)	4.0 (1.5)	4.1 (1.8)	**0.03**°

*Abbreviations used: Smear, sputum smear microscopy; PTB, pulmonary TB; EP-TB, extrapulmonary TB; FNAC,fine needle aspiration cytology; DOTS, directly observed therapy short course.

**Pearson's chi-squared (or Fisher's exact) test for categorical variables comparing chest physicians plus other MD/MS practitioners versus MBBS practitioners in the private sector; mean ISTC score assessed using linear regression.

°Remains significant risk factor (P≤0.05) for ISTC adherence comparing practitioner level of training after adjustment for sex, years practicing, facility type, and total patient volume.

†H = isoniazid, R = rifampicin, Z = pyrazinamide, and E = ethambutol, irrespective of whether the regimen was daily or intermittent.

Ŧ Data for 68 private practitioners who refer all patients with TB for treatment were excluded from evaluation for ISTC8. This standard was excluded from the calculation of the ISTC score.

**Fig 2:**
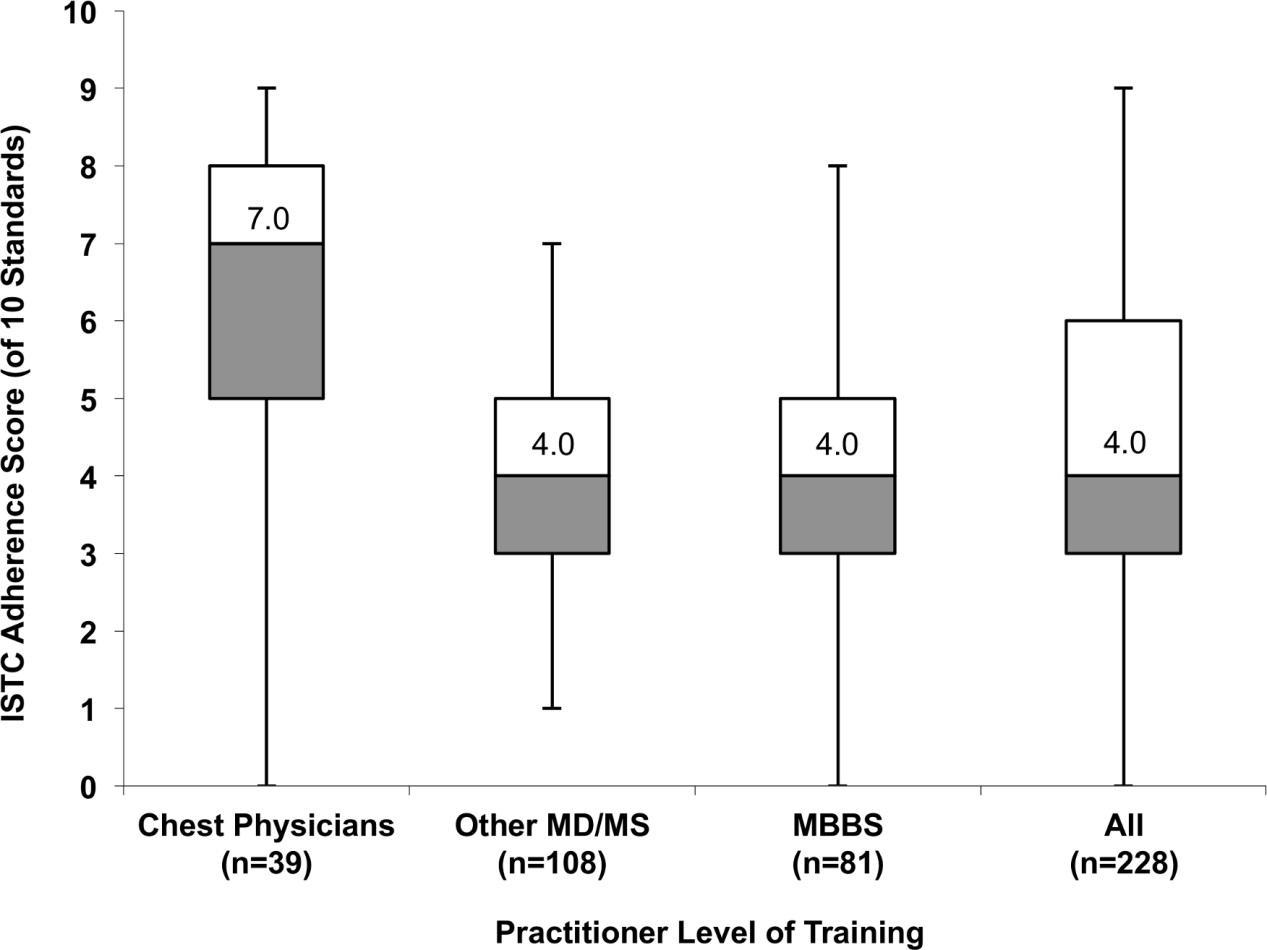
Distribution of aggregate practitioner-reported adherence scores to ten of the International Standards for TB Care by practitioner training in the private sector in Chennai. Practitioner-reported practices were evaluated against 10 of the International Standards for TB Care (ISTC) for which comparative data were collected, and an ISTC score was calculated by summing the total number of standards for which reported practices agreed with ISTC recommendations. For example, a score of seven means the corresponding practitioner reported practices in accordance with seven of the 10 standards that we evaluated. Of 10 standards evaluated, the overall median ISTC adherence score was 4.0 (IQR 3.0–6.0). Chest physicians reported greater adherence than other MD/MS practitioners with higher levels of training (median 7.0 vs. 4.0, P<0.001), or MBBS practitioners (7.0 vs. 4.0, P<0.001). Box plots depict the median (central line), interquartile range (box), and range (whiskers).

Median ISTC scores were significantly higher among chest physicians than other MD/MS practitioners (7.0 vs. 4.0, P<0.001), or MBBS practitioners (7.0 vs. 4.0, P<0.001), reflecting greater reported adherence (Fig 2). Providers practicing in private standalone clinics had significantly lower median ISTC scores than those practicing in private hospitals (4.0 vs. 5.0, P<0.01), and in both government and private healthcare settings (4.0 vs. 5.5, P<0.01). Further, providers that saw more than 12 patients with TB per year had significantly higher median ISTC scores than those who saw fewer patients with TB (5.0 vs. 3.0, P<0.001). There were no significant differences in PPs’ median ISTC score by census tract zone. After adjusting for gender, practice years, and total patient volume, chest specialists had higher mean ISTC scores than MBBS practitioners (mean difference in score: 2.4, 95%CI: 1.7–3.0). PPs practicing in private hospitals and in both government and private healthcare settings had higher mean ISTC scores than those in private standalone clinics (mean difference in score: 1.3, 95%CI: 0.7–2.0; and 0.6, 95%CI: 0.3–1.1; respectively).

Of 228 responding PPs, 160 listed 27 different treatment regimens for PTB, while the other 68 referred all patients for TB treatment. Prescriptions inconsistent with ISTC included incorrect treatment durations (16/160), drug regimens lacking pyrazinamide and/or ethambutol (10/160) or including quinolones or injectable second-line agents (10/160). Of 160 PP’s, only 78% prescribed a regimen consisting of (a) 6 to 8 months of isoniazid and rifampin, (b) at least one of pyrazinamide or ethambutol, and (c) no second-line agents for a hypothetical patient with new PTB. Appropriate prescription practices were significantly lower among chest physicians compared to MD/MS practitioners (54% (20/37) vs. 87% (67/77), P<0.001) and MBBS providers (54% (20/37) vs. 83% (38/46), P<0.01), but not among providers that saw over 12 TB patients per year versus those who saw less (80% vs. 77%). Inappropriate prescriptions among chest physicians included 27% prescribing incorrect treatment durations exceeding six to eight months (vs. 7% other MD/MS and 2% MBBS), 11% prescribing regimens including quinolones or injectable second-line agents (vs. 5% other MD/MS and 4% MBBS), and 8% prescribing regimens lacking pyrazinamide and/or ethambutol (vs. 1% other MD/MS and 11% MBBS).

Only 40% of all PPs utilized DOTS-based approaches to ensure treatment adherence, including referral to RNTCP DOTS (55/228), REACH PPM (30/228), or both (7/228). In adjusted analyses, use of these approaches was significantly among lower chest physicians and other MD/MS practitioners compared to MBBS providers (Table 2). One-third of PPs reported using sputum smear, culture, or Xpert to monitor patients taking PTB treatment. Chest physicians were more likely to assess patients’ response to treatment compared to MD/MS practitioners (69% (27/39) vs. 21% (23/108), P<0.001) and MBBS providers (69% (27/39) vs. 32% (26/81), P<0.001)

## Discussion

Diagnostic and treatment practices were variable in this study of qualified private practitioners who diagnosed at least one patient with TB in the past year in the urban Indian private sector. As examples, less than half of surveyed practitioners tested patients with long-lasting cough for TB, nearly half used TST as part of their suite of diagnostic tests for diagnosis of active TB, less than one-quarter notified patients treated for TB to public health authorities, and over one-fifth used treatment regimens that were either too short, lacked critical drugs, or included second-line agents for patients with new pulmonary TB. NGO-led efforts to engage PPs in PPM partnerships in Chennai [13] have emphasized appropriate TB diagnostic practices, including usage of smear microscopy. Nonetheless, given that most patients with TB in urban India are diagnosed and treated in the private sector, the underutilization of WHO-approved diagnostic tests such as Xpert and low adherence to ISTC highlights the need for widespread engagement of private practitioners for adequate TB control.

Prior studies of practitioners in India have consistently documented variable quality of TB care in the private sector [6,13,19]. India’s RNTCP strives to provide universal access to quality TB diagnosis and treatment for all patients with TB as part of the national strategic plan for 2012–2017 [20]. However, in a recent systematic review, only half of the healthcare providers from both the public and private sectors tested patients with long-lasting cough for suspicion of TB, and two-thirds used smear microscopy for patients with presumptive TB [6]. In comparison, a recent study using standardized patients seeking care for TB symptoms in the private sector found that only 4% of PPs actually ordered any sputum testing versus 45% of practitioners that reported they would order a sputum test for such a patient [21]. Over three-quarters of providers in the systematic review prescribed a treatment regimen potentially consistent with ISTC guidelines for new patients with PTB, higher than in other explorations of private sector TB treatment practices [5,6,9]. The standardized patient study reported correct case management by only 10% of private practitioners for a patient presenting with classic TB symptoms versus 73% by PP self-report [21]. Using practitioner-reported data that may underestimate correct case management in actual practice, our findings provide further evidence suggesting that TB diagnostic, treatment, and notification practices in a randomly recruited sample of urban PPs remain inadequate. These findings are particularly concerning given that India has the world’s highest TB burden, including reports of poor treatment outcomes and emerging drug resistance, especially in urban areas [6,22–24].

Our study is among the first to assess PP practices by patient volume, practitioner specialty, and type of practice facility [6]. We found that chest specialists and those seeing higher volumes of TB patients had somewhat more appropriate diagnostic, treatment, and notification practices, though critical gaps still remain with respect to appropriate prescription and patient-centered treatment approaches, even with efforts by the RNTCP to ensure quality care and make TB a notifiable disease [25,26]. We describe a comprehensive evaluation of diagnostic practices among urban private practitioners, which often included non-microbiological tests (e.g., TST) not generally recommended for active TB diagnosis. Use of TB-specific diagnostic tests was highest among practitioners with higher levels of training, though usage of WHO-approved diagnostic tests, such as Xpert, was minimal at the time of this study. We found a strong association between use of sputum smear microscopy for initial diagnosis and more rapid treatment initiation, though the majority of providers reported sending patients to private laboratories for diagnostic testing, which may result in additional delays in TB diagnosis [4,27]. Additionally, we noted lower ISTC adherence to diagnostic and treatment practices among those practitioners with an MBBS degree, practicing in private standalone clinics, or seeing fewer than 12 patients with TB per year. These findings offer new insights regarding factors associated with suboptimal care among subsets of providers in the private sector that can be targeted by interventions to improve the quality of TB care.

Mismanagement of TB diagnosis and treatment negatively affects the patient and the Indian public health system. The lack of testing for TB among patients with long-lasting cough and the use of inappropriate diagnostic tests for active TB (e.g., TST) by private practitioners in this study may be due to modified practices to make medical visits more convenient to avoid losing patients who may seek fast results or no tests to reduce costs [27]. The use of inappropriate treatment regimens for new patients with TB may stem from attempts to provide instant relief of symptoms, such that private providers favor empiric treatment or prescription of stronger or second-line antibiotics [27]. Additionally, the lack of regulation among private practitioners outside of the RNTCP results in a lack of guidance for TB management practices and may be partly responsible for the poor adherence to national TB guidelines and ISTC [19]. These drivers extend to the practice of patient notification where private practitioners have poor understanding of the process that contributes to the “know-do” practice gap [21,28]. These challenges in the private sector underscore the importance of stronger dissemination, implementation, and enforcement of the ISTC and Standards for TB Care in India for all Indian practitioners.

As a cross-sectional study of PP practices, this study has several important limitations. We included only formally trained qualified practitioners and providers referring to a public-private mix organization. While we were thereby able to attain a high response rate, our results are not representative of all urban private practitioners providing TB care (including unqualified providers and practitioners of alternative health systems) and likely overestimate the quality of care provided in Chennai as a whole. We restricted interviews to PPs who had diagnosed at least one patient with TB in the past year to reduce recall bias. Further, we used structured questionnaires for data collection, which allowed us to collect a larger volume of data in the short times available to interview PPs; however, for describing actual practices, vignette-based questions may be preferred [6]. Lastly, our survey captures PPs’ knowledge and self-reported practices, but not actual practice. A recent study from India using standardized patients to overcome this limitation reported a wide gap between what providers know, and what they do in real practice, suggesting the need for additional empiric data on actual practices [21].

Our findings emphasize the TB burden and management challenges to practitioners in the urban private sector with several implications for policy. First, TB diagnosis and treatment patterns were variable, and while chest physicians and high-volume providers reported greater ISTC adherence in some aspects (e.g., use of microbiological tests), they reported lower adherence in others (e.g., prescription patterns). This widespread variability in practices highlights the need for broad-based education and involvement at all levels of the private sector. Practices in the informal sector and among lower-volume providers are expected to be substantially worse [21]. Second, practitioners who reported the shortest health system delays utilized smear microscopy as the initial TB diagnostic test, demonstrating the importance of performing TB-specific microbiological tests at the first patient visit to decrease diagnostic delay and TB transmission. Finally, as the RNTCP invests in deploying TB diagnostic tests capable of same-day diagnosis such as Xpert, and IPAQT works to increase test availability in private laboratories, mechanisms of public-private cross-referral and regulation are essential to engage PPs and link their patients to guideline-based TB care [12,15]. Modeling analyses suggest that such private-public mechanisms and availability of quality diagnostics can have a substantial population-level impact on TB in India [29].

## Conclusion

In conclusion, our study shows variability in TB diagnostic and treatment practices in India’s urban private sector, including among qualified providers, high-volume providers, chest physicians, and those referring to public-private mix organizations for TB treatment. In particular, screening of individuals with persistent cough, use of TB-specific tests at the initial encounter, expanded use of molecular tests and treatment monitoring, and prescription of appropriate first-line regimens for PTB should be emphasized. Innovative approaches to TB control in India must include broad-based engagement of the private sector if TB elimination in India is to become a reality in the foreseeable future.
